# Immunohistochemical analysis of ezrin-radixin-moesin-binding phosphoprotein 50 in prostatic adenocarcinoma

**DOI:** 10.1186/1471-2490-11-12

**Published:** 2011-06-14

**Authors:** Tanner L Bartholow, Michael J Becich, Uma R Chandran, Anil V Parwani

**Affiliations:** 1 University of Pittsburgh School of Medicine, Pittsburgh, PA, USA; 2 Department of Biomedical Informatics, University of Pittsburgh School of Medicine, Pittsburgh, PA, USA; 3 Department of Pathology, University of Pittsburgh School of Medicine, Pittsburgh, PA, USA

## Abstract

**Background:**

Ezrin-radixin-moesin-binding phosphoprotein 50 (EBP50) is an adapter protein which has been shown to play an active role in a wide variety of cellular processes, including interactions with proteins related to both tumor suppression and oncogenesis. Here we use immunohistochemistry to evaluate EBP50's expression in normal donor prostate (NDP), benign prostatic hyperplasia (BPH), high grade prostatic intraepithelial neoplasia (HGPIN), normal tissue adjacent to prostatic adenocarcinoma (NAC), primary prostatic adenocarcinoma (PCa), and metastatic prostatic adenocarcinoma (Mets).

**Methods:**

Tissue microarrays were immunohistochemically stained for EBP50, with the staining intensities quantified using automated image analysis software. The data were statistically analyzed using one-way ANOVA with subsequent Tukey tests for multiple comparisons. Eleven cases of NDP, 37 cases of NAC, 15 cases of BPH, 35 cases of HGPIN, 103 cases of PCa, and 36 cases of Mets were analyzed in the microarrays.

**Results:**

Specimens of PCa and Mets had the lowest absolute staining for EBP50. Mets staining was significantly lower than NDP (p = 0.027), BPH (p = 0.012), NAC (p < 0.001), HGPIN (p < 0.001), and PCa (p = 0.006). Additionally, HGPIN staining was significantly higher than NAC (p < 0.009) and PCa (p < 0.001).

**Conclusions:**

To our knowledge, this represents the first study comparing the immunohistochemical profiles of EBP50 in PCa and Mets to specimens of HGPIN, BPH, NDP, and NAC and suggests that EBP50 expression is decreased in Mets. Given that PCa also had significantly higher expression than Mets, future studies are warranted to assess EBP50's potential as a prognostic biomarker for prostate cancer.

## Background

Prostate cancer is currently the second leading cause of cancer death in males[1]. Despite this, in the era of prostate specific antigen (PSA) screening, researchers have now estimated that clinically insignificant prostate cancer is actually overdiagnosed at a rate of 29% for whites and 44% for blacks, the PSA screen resulting in the detection of cancers that otherwise would only have been detected during autopsy in up to 15% and 37% of tumors in whites and blacks, respectively[2].

There is currently a limited amount of information in the literature on biomarkers with the potential to discern which cases of prostate cancer have the greatest potential to metastasize versus remain latent[3]. Evaluating the expression of tumor suppressor proteins that have been previously examined in other cancers may indicate novel biomarkers for prostate cancer that have the potential to assess individual patient prognosis and guide therapy selection.

One such biomarker is ezrin-radixin-moesin-binding phosphoprotein 50 (EBP50), which is also known as Na+/H+ exchanger regulatory factor 1, or NHERF1. A 50 kDa, 358 amino acid adaptor protein whose gene is located at 17q25.1, it consists of two PSD-95/Discs Large/ZO-1 (PDZ) domains and a carboxyl-terminal region that is capable of binding members of the ezrin-radixin-moesin (ERM) protein family[4-6]. With its multiple domains, it has been described as a participant in at least 30 unique cellular interactions, including those involving ion transport, secondarily coupled signaling receptors, and tyrosine kinase receptors[5]. Among the oncologically relevant functions for the protein that have been demonstrated are its ability to recruit the tumor suppressor PTEN for inactivation of the phosphatidylinositol-3-OH kinase (PI3K)/Akt signaling pathway in glioblastoma multiforme[5,7], as well as an ability to provide cortical stabilization of β-catenin at cellular junctions in murine embryonic fibroblast models[8], both indicative of a tumor suppressor function.

Further supporting this notion, additional work has shown that an allele for EBP50 was deleted in 28 of 48 examined breast cancer cell lines[9]. Knocking-out existing EBP50 expression in T47D and MCF7 breast cancer cell lines has also been shown to lead to increased cell proliferation[10]. Zheng et al. have additionally noted that restoring EBP50 expression to a MDA-MB-231 breast cancer line, originally deficient in EBP50, inhibited cell growth and increased apoptosis[11]. Subsequent to this, the same group prepared a stably transfected HeLa-EBP50 clone, which also demonstrated decreased cell growth, suggesting a tumor suppressor role for EBP50 in cervical cancer as well.

In spite of this, a universal tumor suppressor function for EBP50 has not been observed. EBP50 has been shown to be overexpressed in hepatocellular carcinoma[12]. Cytoplasmic over expression has also been linked to the progression of colorectal carcinoma[13]. Furthermore, in contrast to the previously described work, Song et al. have reported that EBP50 immunoreactivity in breast cancer was positively associated with tumor stage and lymph node involvement[14], prompting others to suggest that its role in oncogenesis or tumor suppression may vary with cellular location, with a membranous or apical distribution supporting a tumor suppressor function and a cytoplasmic distribution conferring oncogenic properties [5].

To our knowledge, the expression of EBP50 has never been studied in prostate cancer. Here, we compare the immunohistochemical profiles in a series of 11 cases of normal donor prostate (NDP), 37 cases of normal tissue adjacent to prostatic adenocarcinoma (NAC), 15 cases of benign prostatic hyperplasia (BPH), 35 cases of high-grade prostatic intraepithelial neoplasia (HGPIN), 103 cases of primary prostatic adenocarcinoma (PCa), and 36 cases of metastatic prostatic adenocarcinoma (Mets) in order to examine if either a tumor suppressor or oncogenic function for EBP50 can be suggested in prostate cancer, providing further information about its potential as a diagnostic and/or prognostic biomarker.

## Methods

### Tissue Microarray Block Preparation

Tissue microarray (TMA) blocks were constructed using specimens obtained from the Health Sciences Tissue Bank at the University of Pittsburgh Medical Center, with the tissue bank rendering the honest broker services. All specimens were originally obtained with informed consent. Cores from the appropriate case specific paraffin-embedded tissue blocks were assembled into TMAs as described in a previous protocol[15]. The final TMAs consisted of 11 cases of NDP, 37 cases of NAC, 15 cases of BPH, 35 cases of isolated HGPIN (no accompanying cancer diagnosed), 103 cases of PCa, and 36 cases of Mets. No specimens of HGPIN included in this study were diagnosed at the time as containing PCa. All cases were initially prepared so that each one would be represented at least in triplicate. Due to variations in TMA processing, however, some cases were only able to be represented in duplicate. This occurred for three cases of the HGPIN, three cases of the Mets, three cases of the NAC, two cases of the BPH, and eight cases of the PCa. In such instances, these cases were still scored and included as a part of the final analysis.

### Immunohistochemistry

Each TMA block was deparaffinized and then rehydrated with incremental ethanol concentrations. Decloaker was then used for heat induced epitope retrieval, followed by a 5 minute TBS buffer rinse. A Dako autostainer was then used to stain the TMAs with anti-EBP50 (working dilution 1:400), a mouse monoclonal antibody (Catalogue # MA1-19291) from Thermo Scientific (Waltham, MA). Immunolabeling was conducted using Dako Dual Envision + Polymer (Catalogue # K4061) from Dako (Carpinteria, CA). The slides were counterstained with hematoxylin and coverslipped.

### Scoring of Slides

All slides for this project were scanned as digital whole slides images (WSI) using ScanScope XT (by Aperio, Vista, CA). The individual tissue cores for each WSI were viewed using Aperio ImageScope (Version 11.0.2.716) and scored by applying the Positive Pixel Count Algorithm to each one. In order to detect the EBP50 staining, a hue value of 0.1 and hue width of 0.5 was chosen for the algorithm, corresponding to the suggested range for the detection of brown immunostaining using the software (Aperio Positive Pixel Count Algorithm instruction manual). By analyzing the average pixel intensity with a predetermined hue value and width, the stromal tissue and cell nuclei that appear blue and do not feature the immunostain are negated by the software and excluded from the final analysis that determines the average staining intensity. This, in effect, controls for the glandular to stromal tissue ratio present in the TMA cores.

The validity of using Aperio software for quantitative immunohistochemistry has been previously documented in other studies[16,17]. The average staining intensity was then determined by the software for each core, utilizing a formula that sums the intensities of weak, moderate, and strong staining pixels and divides this value by the total number of weak, moderate, and strong pixels. Staining intensities for the software are reported on a scale of 0-255, corresponding to light transmission through the specimen. Therefore, higher staining intensities correspond with lower scores on the light transmissibility scale. Scores in the range of 220-175 are classified as weak staining, 175-100 are classified as moderate staining, and 0-100 are classified as strong staining. In order to make staining scores more intuitive in our figures, our results are reported as the difference between no stain detection (255) and the average staining intensity as reported by the software, so that higher values correspond with the higher staining intensities. This value is referred to as the "staining intensity" throughout the rest of the manuscript.

The means for each case, and subsequently for each tissue type were then determined. For the specimens of adenocarcinoma, the Gleason score and tumor stage, where available, were also reported. The Clinical TNM, as opposed to the Pathologic TNM, staging classification was used to assess the specimens. All means were reported with standard errors.

One-way ANOVA with subsequent Tukey tests for multiple comparisons (α = 0.05) were used to compare the tissue types, PCa carcinoma stages, and PCa Gleason scores. Graphical analysis was conducted using Microsoft Excel 2007 (by Microsoft Corporation, Redmond, WA) and R: A Language and Environment for Statistical Computing (R Development Core Team. R Foundation for Statistical Computing Vienna, Austria. 2011. ISBN 3-900051-07-0, <http://www.R-project.org>).

Photomicrographs of tissue cores were taken using an Olympus BX51 microscope using Spot Advanced V4.6 (Diagnostic Instruments, Inc.) software. All images were taken at 20×.

This study received exempt approval (PRO08040368) from the University of Pittsburgh Institutional Review Board.

## Results

### Patient Ages

The average patient ages with standard deviations for the tissue types in this study are NDP 32 ± 13 years, NAC 63 ± 6 years, BPH 67 ± 9 years, PIN 63 ± 8 years, PCa 64 ± 9 years, and Mets 70 ± 10 years.

### Staining Intensities

**Figure 1:**
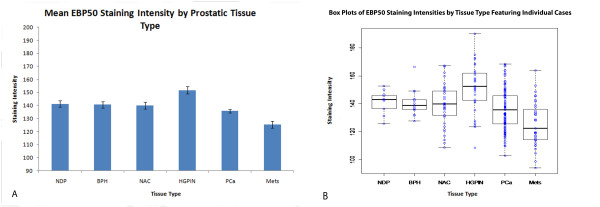
**EBP50 staining intensity by prostatic tissue type**. A) Mean EBP50 staining score by prostatic tissue type. Significant differences were seen between Mets and NDP (p = 0.027), NAC (p < 0.001), BPH (p < 0.012), PIN (p < 0.001), and PCa (p = 0.006), with Mets having the lowest staining of any group. PIN also had the highest staining of any group, and was significantly higher than PCa (p < 0.001) and NAC (p < 0.009) groups. B) Box plots of EBP50 Staining Intensities by Tissue Type Featuring Individual Cases. It is especially notable that many specimens in the PCa and Mets classifications feature staining below that of the other tissue classifications. Moreover, only one case of Mets featured a score above 155, the lowest limit for high intensity staining.

The mean staining scores for NDP, BPH, NAC, HGPIN, PCa, and Mets were 141.23 ± 2.43, 140.66 ± 2.42, 139.91 ± 2.52, 151.76 ± 2.88, 135.72 ± 1.45, 125.55 ± 2.63 (Figure 1A). Box plots showing the individual staining scores are featured in Figure 1B. A one-way ANOVA (p < 0.001), with subsequent Tukey tests for multiple comparisons, showed significant differences between Mets and NDP (p = 0.027), NAC (p < 0.001), BPH (p = 0.012), HGPIN (p < 0.001), and PCa (p = 0.006). Differences were also seen between HGPIN and PCa (p < 0.001) and NAC (p < 0.009).

Five out of thirty-seven specimens of NAC (13.5%), 1/15 specimens of BPH (6.7%), 17/35 specimens of HGPIN (48.6%), 10/103 specimens of PCa (9.7%), and 1/36 specimens of Mets (2.8%) had staining scores in the highest intensity category. Although 0/11 specimens of NDP had staining in the highest intensity category, 4/11 specimens were less than 10 intensity scale points below the threshold for highest intensity category (36.4%).

When classified by tumor stage, the mean scores were stage 2, 139.60 ± 2.43 (n = 39), stage 3, 130.66 ± 2.29 (n = 37), and stage 4, 137.03 ± 2.61 (n = 27). A resultant one-way ANOVA (p = 0.024) with subsequent Tukey tests for multiple comparisons showed significant differences between stage 2 and stage 3 (p = 0.021).

When classified by Gleason score, the average staining score was 133.13 ± 3.52 (n = 15) for those with a score of 6 or less, 135.69 ± 2.06 (n = 49) for those with a score of 7, and 137.13 ± 2.54 (n = 38) for those with a score of 8 or more. No significant differences were seen between the Gleason score classifications (p = 0.672).

### Staining Patterns

**Figure 2:**
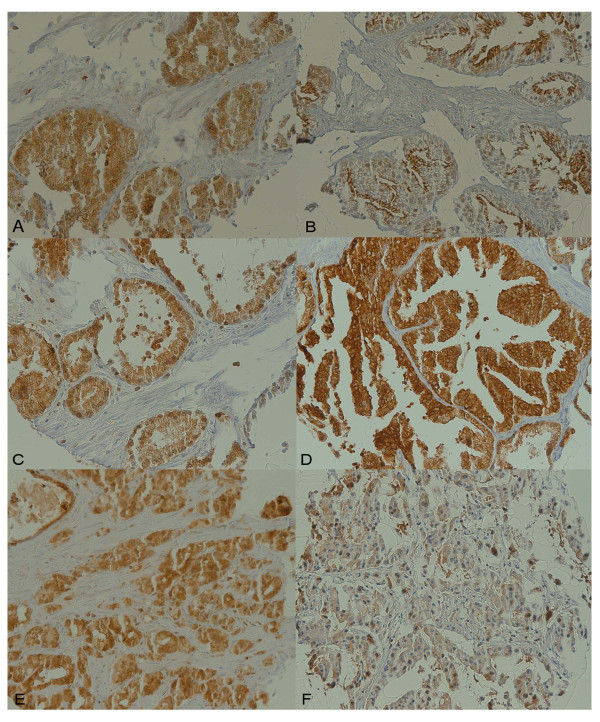
**Photomicrographs of TMA cores**. Photomicrographs of TMA cores (20×). A) NDP, B) BPH, C) NAC, D) HGPIN, E) PCa, and F) Mets. Note the predominantly membranous pattern in BPH, which was noted in cores from 11/15 cases. To a lesser degree, this is also noted in the depicted specimen of HGPIN, although the cytoplasmic staining is also intense, which partially obscures the distinction. In the remainder of depicted cores, the staining is largely cytoplasmic. No membranous staining was noted in any of the metastatic cores.

Representative photomicrographs of the TMA cores are shown in Figure 2. Across most specimens, a cytoplasmic EBP50 staining pattern was noted. However, in many specimens, more prevalent amongst the benign and pre-cancerous tissues, a membranous or apical staining pattern was distinctly notable as more predominant than co-accompanying cytoplasmic staining. Such cores were noted in 3/11 cases of NDP (27.3%), 11/15 cases of BPH (73.3%), 12/37 cases of NAC (32.4%), 12/35 cases of HGPIN (34.8%), 10/103 cases of PCa (9.7%), and 0/36 cases of Mets.

## Discussion

In the EBP50 stained specimens, the average staining intensities were highest in the specimens of HGPIN, and lowest in the specimens of PCa and Mets. HGPIN had significantly higher staining than Mets, NAC, and PCa. Despite the fact that HGPIN was significantly different than the BPH and NDP groups but not the NAC group, the means were relatively similar between BPH, NDP, and NAC, suggesting that the staining intensity does not vary greatly between these classifications.

Previous work has shown that radixin, an ERM protein and binding partner of EBP50 that is responsible for linking F-actin to plasma membrane proteins[5,18], also demonstrated higher absolute staining in specimens of HGPIN than in other prostatic tissue types, including PCa and NAC [19]. This finding may indicate that the higher expression of both proteins in HGPIN may reflect a unique feature of the pre-cancerous tissue physiology, and that both may be down regulated in specimens of prostatic adenocarcinoma.

Mets tissue had significantly lower staining than all other tissue types, including PCa, indicating that loss of EPB50 expression may play a role in select cases of prostate cancer metastasis. This is further echoed by the fact that only 1/36 cases of metastatic tissue showed high intensity range staining for EBP50. Given that EBP50 has been previously shown in murine fibroblast models to promote adherens junction stabilization through mediating the interaction of β-catenin with E-cadherin, its loss of expression is plausible with tumor dissemination[8].

Despite this, however, it is important to note that these findings may also contain a correlative component that is not prognostic in nature. While all of the tumors in this study were primary tumors at the time of specimen retrieval, definitive follow-up information on these patients was not available. In this sense, from this study it is not possible to rule out that the decreased EBP50 expression, at least in part, may be due to the metastatic location itself. The current results, however, indicate and warrant later phase biomarker studies[20] that will longitudinally correlate EBP50 expression directly with patient outcomes to further evaluate its potential to predict metastatic risk in prostate cancer.

A significant increase in EBP50 staining between Stage 2 and 3 PCa specimens was also observed. It is possible that this finding represents a change in tumor physiology between the two stages, however, given that this trend was not noted in Stage 4 PCa specimens, it is also possible that this represents a spurious finding, and should be further evaluated before definitive conclusions are reached. No differences were seen between the Gleason score classifications.

In general, EBP50's expression is increased in polarized epithelial cells, such as the liver, kidney, pancreas, small intestine, and the prostate[5]. Considering the diverse intracellular roles that have been proposed for EBP50, it may be difficult to elucidate a clear, singular mechanism whereby it may promote oncogenesis or tumor suppression within individual tissue types.

Differing hypotheses to explain to the behavior of EBP50 in cancer have been proposed, especially given the multiple studies that seem to support two opposing functions for EBP50 in cancer [9-11,21]. Zheng, et al have suggested that in many cases of breast cancer where EBP50 is expressed, it may not be expressed in sufficient quantities to halt tumor progression[11]. Others have demonstrated that the hypoxia associated with tumor necrosis can increase EBP50 (NHERF1), which increases Na+/H+ activity, in turn decreasing local pH and promoting tumor dissemination[4,21]. And while it has been shown that EBP50 can cluster with EGFR, PDGFR, and the tumor suppressor NF2 to halt cell signaling and hence cancer progression[11,22-24], others have posited that EBP50's PDZ domains may actually allow for new tumor-specific interactions[4].

These different findings, in part, have been reconciled with the hypothesis that EBP50 may have different functions by cellular location, with a tumor suppressor function associated with a membranous/apical distribution, and an oncogenic function promoted by a cytoplasmic location[5]. In support of this hypothesis, previous work has demonstrated a progression from luminal to cytoplasmic EBP50 expression occurs across normal to ductal carcinoma in-situ to invasive and metastatic breast cancer tissues[25]. While membranous EBP50 has been shown to stabilize β-catenin at cell membranes[8], non-stabilized β-catenin is also capable of forming growth-promoting transcription complexes in the nucleus and has been prominently associated with hepatocellular carcinoma[26]. Hence, it is interesting that an overexpression of EBP50 with a focal nuclear localization has been documented in hepatocellular carcinoma[12]. A similar phenomenon has been noted in colorectal cancer, where membranous EBP50 loss and increased cytoplasmic expression has been noted in the colorectal adenoma-to-carcinoma transition, with subsequent increases in cellular invasion and epithelial-to-mesenchymal transition, processes that demonstrated reversibility when EBP50 was reexpressed at the apical membrane of intestinal epithelium[13]. Additionally, in normal astrocytes, EBP50 has demonstrated a membranous distribution, while it has demonstrated a cytoplasmic distribution in many cases of glioblastoma multiforme[7]. This corresponds with the absence of the EBP50-binding tumor suppressor PTEN and the activation of the growth-promoting Akt pathway, which is traditionally silenced by PTEN through recruitment to the plasma membrane by EBP50[7].

Relating these examples specifically to prostate cancer, it has been demonstrated that β-catenin can interact with androgen receptor and increase its transcriptional activity, hence contributing to prostate cancer progression[27,28]. Moreover, the tumor suppressor PTEN is frequently found mutated in prostate cancer, with subsequent PI3K/Akt signaling shown to promote cell survival[27,29]. Interestingly, the PI3K/Akt pathway also increases the stability of β-catenin in prostate cancer[30]. Based on these findings, it is possible to suggest that EBP50, under the appropriate circumstances, may possess a tumor suppressor function in prostate cancer similar to those described above in other cancer types.

While a cytoplasmic staining pattern for EBP50 was noted across most specimens examined in this study, a membranous/apical staining pattern was clearly more prominent than cytoplasmic staining in many cores, most commonly in the benign and pre-neoplastic specimens (Figure 2). While cores with clearly more prominent membranous staining were found in 73.3% of BPH cases studied (Figure 2B), this finding was only noted in 9.7% of cases of PCa and not in any specimens of Mets. Although an overlap between the expression patterns still existed between many of the benign and cancerous specimens, this trend concurs with the above hypothesis regarding strong membranous expression and tumor suppression and warrants further study to determine its potential to assess metastatic risk.

As a final note of interest, in one model of EBP50 function, its PDZ-2 domain has been shown to bind to the C-terminal ERM-binding domain, inhibiting the binding of other proteins to the PDZ domains, such as PTEN and B-catenin[31]. In this same model, when ezrin, an ERM protein, binds the C-terminal domain, the PDZ domains are freed up for additional binding partners. In the intestinal epithelium of ezrin knock-out mice, EBP50 has been shown to be displaced to the cytoplasm[5,32]. In light of this, it is an interesting finding that ezrin expression has been inversely correlated with tumor differentiation in prostate cancer[33], and that moesin, another ERM protein, showed higher incidences of lymph node metastases when it was associated with a cytoplasmic distribution as opposed to a membranous one in oral squamous cell carcinoma[34]. Hence, EBP50's location and function may also be directly linked to the location and presence of the ERM proteins that ultimately enable PDZ domain interactions.

## Conclusions

These results provide a basis for the characterization of the staining patterns and intensities of EBP50 in PCa and Mets in comparison to benign prostate tissue. The immunostaining was highest in specimens of HGPIN. Its expression was lowest in Mets, with the expression in Mets significantly lower than that of all other tissue groups, including PCa (p = 0.006). As a significant decrease between Stage 2 and 3 cancer was observed (p = 0.021), it is also possible that EBP50 expression is altered with carcinoma stage, although significant differences were not seen when Stages 2 and 3 were compared to Stage 4. No differences were seen when comparing the Gleason scores of the PCa specimens.

EBP50 staining was a combination of membranous/apical and cytoplasmic in the prostatic tissues examined. Although a clear distinction would not be made in all cases, a predominant membranous staining pattern was more commonly observed in benign specimens than in malignant ones. As an example, 11/15 cases of BPH featured cores with a readily apparent predominant membranous staining pattern compared to the adjacent cytoplasm, while this was only observed in 10/103 cases of PCa and was not seen in any cases of Mets.

It is also important to note, however, as this membranous/cytoplasmic distinction was not noted in all comparisons between benign and cancerous specimens, that an alternate explanation may account for these findings. As a general decrease in overall staining intensity was observed between the benign specimens and the cancerous/metastatic specimens, this lack of membranous staining may simply reflect an overall staining decrease, making it difficult to appreciate the true ratio of membranous to cytoplasmic staining from a visual examination. This is especially true in the cases of metastatic cancer, where the overall staining is very faint across all specimens in the first place.

Given that a significant decrease in staining was noted between specimens of PCa and Mets, further studies of EBP50 are justified to assess its potential for clinical usage in prognosis assessment of patients with prostate cancer.

## Competing interests

The authors declare that they have no competing interests.

## Authors' contributions

TB assisted in scoring tissue microarrays under the direct supervision of an attending pathologist, performed the statistical calculations, and drafted the manuscript. UC assisted with statistical calculations and reviewed the manuscript. AP conceived of the study, developed and approved the study protocol, approved all tissue microarray scoring, and revised the manuscript. MB also conceived of the study, developed and approved the protocol, and revised the manuscript. All authors have read and approved the final manuscript.

## Pre-publication history

The pre-publication history for this paper can be accessed here:

http://www.biomedcentral.com/1471-2490/11/12/prepub
